# Current Hospital Topics

**Published:** 1907-02-23

**Authors:** 


### HOSPITAL ADMINISTRATION.

### CONSTRUCTION AND ECONOMICS.

### CURRENT HOSPITAL TOPICS.

#### The Annual Meeting.

We have pleasure in announcing that the annual Court of Governors of King's College Hospital will be held at the hospital, Portugal Street, W.C., on February 28th at 4 o'clock. The Royal Free Hospital's meeting is on February 25th at 4.15; and that of the Cancer Hospital at 4 P.M. on February 27th. We shall be very glad to announce the date and hour of the annual meetings of the principal hospitals, if due notice be sent to us a fortnight before the date fixed. In present circumstances notices are frequently sent so late as to make it impossible to announce the date in THE HOSPITAL. This week, for instance, we have received three notices, one within 24 hours, and two others within six days, of the date of the meeting. We mention the matter because we are always glad to co-operate with the secretaries by encouraging the attendance of governors, with the object of making the annual meeting an event in the hospital year. It ought then to be attended by increased popularity, and so made the means of producing additional funds.

#### Hospital Reconstruction in London.

We have had the pleasure of making a careful inspection of several portions of the Royal Free Hospital in Gray's Inn Road. In the ten years which have elapsed since we last inspected this hospital many and great improvements have been made. The buildings are, of course, not new, but great ingenuity and skill have been exhibited in introducing modern improvements by utilising every inch of space to the fullest extent. The result is that, so far as the wards are concerned, the necessity for rebuilding has been obviated for practical purposes, and that the full requirements of a modern hospital will be supplied when a new out-patient department, with accommodation for certain special departments, and new and modern operation theatres are provided. We feel confident that the committee would be well advised to instruct the architect to prepare plans with estimates for the whole of these buildings, and then to set to work and raise the money. The actual sum involved in the modern sense is not excessive. We have no doubt that it would be readily forthcoming if the right steps were taken to secure this result. Any visitor of experience must be pleased with the stamp of the practical character of the work being done, which the whole institution bears, as is evidenced by the actual condition of the wards and other portions of the hospital. We congratulate the authorities upon the present high efficiency of the whole establishment, which has greatly advanced under the treasurership of Mr. Chas. Burt, who has rendered excellent service to the institution in many ways during a long period of years.

#### Some Cruelties of the Voting System.

"E. W." a candidate for a pension at the British Home and Hospital for Incurables, Streatham, S.W., has circulated a letter and correspondence between himself and the secretary, which, assuming the statements are correct, graphically illustrates one aspect of the cruelties entailed upon poor candidates by the voting system. "E. W." was selected as a candidate for pension in September 1906, and in his efforts to be elected in the following November spent £5, by which means he secured 34 votes. This expenditure seems to have exhausted "E. W.'s" resources, and on the 26th January he wrote to the secretary at 72 Cheapside, E.C., to inform him that he was compelled to apply for parish relief. He expresses much distress at this circumstance, due to the fact that "I am quite at the end of my resources, and my wife and children are suffering owing to my inability to provide for their needs." It appears that rule 32 of the Streatham institution provides, in effect, that the names of selected candidates receiving parish relief before election will be removed from the voting list. This may be proper enough for certain cases, but should not apply where, as in "E. W.'s" case, the pecuniary exhaustion can be claimed to be directly caused by the voting system. "E. W." appeals to the secretary for consideration in the specially distressing circumstances of his particular case, and the secretary states his opinion that "E. W." is wise under the circumstances in applying for parish relief. He intimates, however, that the rules must be enforced, and that "E. W.'s" name has in consequence been struck out. How any responsible committee can support a system which entails hardships of this description, and perpetuates evils like those just explained, is a mystery for which it is not easy to account. The report of the Charity Voting Reform Association, just issued, gives several cases where voting charities have reformed their system so as to prevent cruelties of this description. This Report shows very clearly, that no real, financial or other justification can be urged, in support of the perpetuation of the old and flagrant evils of the voting system. It ought no longer to be permitted in connection with any modern charity which aspires to be considered well administered.

### The Right and Wrong View.

Much of the excessive free medical relief granted by the hospitals is due to prejudice based upon tradition. The tradition held by many members of the medical staff of the hospitals, as well as by lay officials, is that to survive, as a voluntary institution, a hospital must show an increase in the work done every year. This view, so far as the honorary medical officers are concerned, rests upon the opinion that if patients are discouraged from coming to a hospital, by the enforcement of regulations, tending to reduce the numbers of patients actually admitted to treatment, the numbers will gradually fall off until there are not enough patients to maintain the hospital's reputation, and to provide material for teaching purposes. The view of the lay official, on the other hand, is based upon financial considerations. He dreads the effect upon the giving public of finding that his hospital actually relieved fewer patients in the current than in the preceding year. In fact, both views are mistaken, if the best interests of voluntary hospitals are to be considered in the present day. The reason is that a hospital which restricts its number of patients must improve the quality of its medical service, and so prove most attractive to patients who are really ill. Similarly, now that the larger and more intelligent givers to hospitals have come to take, as they do take, an individual interest in the work done and the cost of that work, any voluntary hospital, which has had the courage to cut down the number of the patients, and to show at the same time that no abuse could exist under the system by which these results had been secured, must in a financial sense benefit very materially. Wrong views, too, are often held as to expenditure. An amusing example of this is afforded by the committee of the Kingston (Hereford) Cottage Hospital, which has just issued a statement referring with some gratification to the fact that the hospital is so valuable because "more dressings and medical appliances have been used." The experienced administrator watches the expenditure, especially upon dressings, with an eagle eye, and any increase above the average indicates to him that something is wrong, not that the hospital is becoming more valuable because more money is being spent on this or that item!

